# Exogenous Melatonin Spray Enhances Salinity Tolerance in *Zizyphus* Germplasm: A Brief Theory

**DOI:** 10.3390/life13020493

**Published:** 2023-02-10

**Authors:** Riaz Ahmad, Meryam Manzoor, Hafiza Muniba Din Muhammad, Muhammad Ahsan Altaf, Awais Shakoor

**Affiliations:** 1 Department of Horticulture, The University of Agriculture, Dera Ismail Khan 29220, Pakistan; 2 Department of Horticulture, Bahauddin Zakariya University, Multan 60800, Pakistan; 3 College of Horticulture, Hainan University, Haikou 570228, China; 4 Teagasc, Environment, Soils and Land Use Department, Johnstown Castle, Co., Y35 Y521 Wexford, Ireland

**Keywords:** ion homeostasis, metabolic activities, antioxidant defense mechanism, brackish water

## Abstract

Fruit orchards are frequently irrigated with brackish water. Irrigation with poor quality water is also a major cause of salt accumulation in soil. An excess of salts results in stunted growth, poor yield, inferior quality and low nutritional properties. Melatonin is a low molecular weight protein that shows multifunctional, regulatory and pleiotropic behavior in the plant kingdom. Recently, its discovery brought a great revolution in sustainable fruit production under salinity-induced environments. Melatonin contributed to enhanced tolerance in *Zizyphus* fruit species by improving the plant defense system’s potential to cope with the adverse effects of salinity. The supplemental application of melatonin has improved the generation of antioxidant assays and osmolytes involved in the scavenging of toxic ROS. The tolerance level of the germplasm is chiefly based on the activation of the defense system against the adverse effects of salinity. The current study explored the contribution of melatonin against salinity stress and provides information regarding which biochemical mechanism can be effective and utilized for the development of salt-tolerant germplasm in *Zizyphus*.

## 1. Introduction

Minor fruit crops are a major source of minerals, vitamins, fiber, proteins, antioxidants and carbohydrates necessary for a healthy life. Among these, *Zizyphus* is a rich source of ascorbic acid compared with other fruits. Therefore, it is famous as a poor man’s apple because of its higher nutrition, cheap prices and easy accessibility at markets. The population of the world is currently 7.7 billion, and this is expected to rapidly increase in the future to nearly 9.7 billion by 2050 [1]. It is necessary to produce food for healthy life within the country. It is time to focus on crops which produce maximum output even with the application of minimum inputs. The focus on underutilized fruit crops is the best way to feed a huge population [2]. The consumption of *Zizyphus* fruits provides healthy life due to their excellent nutritional properties; however, their cultivation is still poor because of poor agricultural lands [3]. Climate change, biotic and abiotic stresses are becoming more challenging for the production of fruit crops. Irregular rains and winds have disturbed the productivity of fruit trees. Irrigation with poor quality and brackish water is a major cause of accumulation of salts within the arable lands. Water problems are associated with industrial effluents and urbanization in developing countries. The bombardment with agro-chemicals in order to obtain higher yields have adversely affected the fruit quality and disturbed food safety, and these are even threats to an eco-friendly environment. For sustainable fruit production, the application of eco-friendly treatments is effective in maintaining fruit quality and protection from environmental hazards [4]. Approximately 20% yield losses have been estimated in the biosphere because of the excessive amount of salts in the soil [1]. 

Accumulation of salts in the root zone of *Zizyphus* trees restricts quality fruit production because of the translocation of salts and toxic ions from the roots to other tree parts. Restriction in the uptake of water and mineral nutrients results in osmotic stress conditions [5]. Nutrition imbalances and water-deficit conditions due to osmotic stress caused stunted growth of seedlings, low yield and poor-quality fruit to persist on trees. However, fruit dropping can also occur due to nutritional imbalances in *Zizyphus* fruit crops [1]. Fruit tree metabolism is also disturbed by excess salts within tree cells, organelles and compartments. Sherani et al. [6] assessed that disturbance in metabolism in different *Zizyphus* cultivars, i.e., Suffan, Mehmud Wali, Karela and Delhi White; these were recorded because they were growing under induced-salinity stress environments. Higher concentrations of salts within tree cells and compartments via the higher uptake, translocation and accumulation of ions may reach toxic levels. Induced-reduction behavior in CO_2_ diffusion, chlorophyll contents, carbohydrate buildup and photosystem II ultimately adversely affects the stomatal regulation. Photosynthetic pigments are also ruptured due to irregular conductance of the stomata because of higher NaCl concentrations [7]. Accumulation of different sugars, phenolic compounds and ascorbic acid are also disturbed due to higher levels of salts in the *Zizyphus* crop. The nutritional profiling of *Zizyphus* fruits is drastically damaged from excessive salt levels. *Zizyphus* plants face oxidative stress conditions due to the over-generation of ROS within plant cells, compartments and organelles. Optimum production of ROS is non-toxic and supportive in tree growth and developmental phases [8]. 

Different factors such as mineral nutrients, molecular basis, proteomics, transcriptomics, metabolites and genomic variability can be utilized to cope with the adverse effects of salt stress in *Zizyphus* crops. Fruit trees activate their defense system to scavenge these toxic ROS. However, ROS is an important stress indicator which determines the intensity of abiotic stresses [9]. Moreover, H_2_O_2_, MDA and lipid peroxidation also indicate the stress intensities [10]. Activation of the defense mechanism determines the tolerance/sensitivity level of *Zizyphus* trees growing under saline conditions, as reported by Li et al. [11] and Back et al. [12]. Little information regarding fruit tree responses under salinity is available, and knowledge about the use of melatonin spray against salinity is still limited in minor fruit crops, especially *Zizyphus*. 

Melatonin is a growth-promoter molecule with numerous diverse impacts in plants. It is effective for improving germination of seeds, root proliferation, flowering, fruit setting, fruit development, fruit ripening and shelf-life [13]. Moreover, postharvest fruit quality is maintained to extend the shelf-life of fruits by application of melatonin [14]. It has excellent scavenging potential against reactive oxygen species (ROS) and reactive nitrogen species (RNS) [15]. Adverse effects of different abiotic stresses, i.e., salinity [16], drought [17,18], heat [19] and cold [20], and biotic stresses, i.e., viruses and bacteria [21,22], can be mitigated by the exogenous application of melatonin. It is also effective at increasing endogenous melatonin levels to cope with harsh climatic conditions. Melatonin is a good way to avoid the toxic effects of heavy metals and pesticides residues present in the produce [23]. Melatonin is a aphytohormone which contributes to the improvement of yield and excellent quality of fruits. 

Different management practices, e.g., molecular breeding, seed priming, agronomic implications, grafting, rhizobacteria, bio-stimulants, and phytohormones, can be employed for the sustainable productivity of crops. Among these, phytohormones, e.g., melatonin, salicylic acid, branosteroids, polyamines, ascorbic acid, jasmonic acid, ethylene, and abscisic acid, are rapid stress-relieving bioactive molecules against stressful conditions. Among these phytohormones, melatonin is an emerging molecule against salinity for exogenous application on fruit crops [5].

Plant researchers are focusing on sustainable fruit production; therefore, appropriate management implications should be explored for enhanced productivity of *Zizyphus* crops, even those growing in saline lands. Exogenous application of melatonin is a very promising approach to cope with saline conditions and enhance tolerance mechanisms in *Zizyphus* fruits. However, the application of melatonin is still unclear, and its effects on *Zizyphus* crop yield, quality and nutritional aspects in salinity-induced environments are poorly understood. The current study encourages and explores the significance of melatonin on the minor fruit crop *Zizyphus* species with regard to its yield, quality and shelf-life in salinity-induced conditions.

## 2. Response and Adaptation Mechanism under Salinity Stress

### 2.1. Melatonin and Exclusion of Na^+^ and Cl^−^

Plants grown in salty zone naturally are halophytes. These plants survived well in 30–500 mM NaCl concentrations [24]. Excellent root hairs and salt-secretion glands are major aspects due to which halophyte plants survive well under saline conditions. The concentration of accumulated salts remains under threshold levels in their plant leaves, as studied by Soni et al. [10]. These plants are considered to be salt-tolerant due to their efficient utilization of Na^+^ and Cl^−^ taken up via roots from saline soil and brackish water. Halophytes are more tolerant compared with other plants due to their xerophytic properties. *Zizyphus* belongs to the Rahmnaceae family. Plants of this family mostly grow in arid and semi-arid regions. Therefore, an excess of Na^+^ and Cl^−^ in the short term at the seedling stage, and long term in higher plants, is toxic by reducing seedling growth, yield and quality of *Zizyphus* fruits [25].

Exclusion of salts is an important inhibitory mechanism to prevent entry of Na^+^ and Cl^−^ into vascular bundles of fruit trees. Na^+^ and Cl^−^ were not accumulated at toxic levels in fruit tree leaves because of the salt-exclusion process [26]. Generally, an excess of salts remains bound in the root zone and stem-basal part of the rootstock because of a mechanism which excludes toxic ions. Moreover, translocation of toxic ions to other tree parts is restricted. Hence, it is an effective mechanism for improving salinity tolerance in fruit trees. Rootstock of wild species like *Zizyphus rotundifolia* are capable toxic ion exclusion compared with other cultivated species. Wild species should be utilized in further breeding programs to develop salt-tolerant germplasm, focusing on fruit yield and quality to feed huge populations [26]. 

Ploidy level is an essential genetic aspect of fruit trees helpful for plants’ survival against adverse climatic conditions. Ploidy in fruit species has greater involvement to cope with adverse effects of salinity and numerous other harsh environmental conditions [27]. In some fruit crops, it has been observed that tetraploid seedlings of citrus exhibited greater salinity resistance behavior compared with diploid ones, as reported by Gao et al. [28]. Moreover, accumulation of Na^+^ and Cl^−^ was found to be higher in tetraploid rootstocks than economic threshold levels compared with diploid rootstocks. However, uptake and accumulation of K^+^ was found to be lower in diploid than tetraploid rootstocks [29].

Accumulation of Na^+^ and Cl^−^ was found to be maximum in the root zone and then in leaves. The salinity-tolerance mechanism is chiefly based on the accumulation and translocation of Na^+^ and Cl^−^ in different portions of the fruit trees [30]. Exogenous application of melatonin had a greater contribution in reducing the accumulation of Na^+^ and Cl^−^ in leaves of fruit crops. The increase in endogenous melatonin improved the plant defense system to uptake and translocate selective ions by excluding toxic compounds [31]. Moreover, an exogenous melatonin spray had the potential to mitigate adverse effects of salinity by alleviating the tree tolerance system against adverse effects of salinity. This phytohormone had an excellent ability to improve the plant’s selectivity for uptake of K+, which is necessary for the proper functioning of tree cells and organelles [32]. Alterations in the activation of the plant defense system against salinity stress is listed in Table 1. The disturbance in fruit quality of *Zizyphus* against salinity is well described (Table 2). The critical concentration of salts that affects the fruit production of *Zizyphus* germplasm is shown in Table 3.

### 2.2. Role of Graft Union against Salinity

Graft union is the combination of rootstock and scion of two diverse genotypes with compatible behavior. Rootstock proves a better anchorage to trees, and most fruit cultivars are grafted in Pakistan [54]. Two well-known species of *Zizyphus*, i.e., *Z. mauritiana* L. and *Z. jujuba* Mill, are commercially grown in Pakistan [3]. Grafting plays a good role in the development of new cultivars with desired traits [55]. Hence, *Z. rotundifolia* is used as a rootstock in Pakistan, while *Z. mauritiana* L. and *Z. jujuba* Mill are used as scions [3]. Moreover, *Z. rotundifolia* is more famous as a hardy species than the other two species, *Z. mauritiana* L. and *Z. jujuba* Mill, with regard to salinity stress conditions [56]. Graft union can inhibit the uptake and translocation of toxic ions from roots toward other parts. Rootstock had a greater contribution in increasing tolerance in grafted cultivars against harsh climatic conditions. Excess amounts of salts accumulated in the root zone of plants may possibly be due to their pre-existence in the soil and also occurs from continuous irrigation with poor quality water from canals and tubewells [1]. It can be assumed that *Z. rotundifolia* as a rootstock can inhibit the uptake and transportation of toxic ions from roots to leaves. The excess of Na^+^ in the root zone may possibly reduce mineral uptake, as studied by Mao et al. [57]. Rootstock revealed better involvement in provision of excellent potential to scion cultivars for higher yield with better desired quality traits. Rootstock plays a major in enhancing the salt-tolerance mechanism in scion cultivars due to inhibition of Na^+^ and Cl^−^ uptake from roots [58].

### 2.3. Melatonin and Root Architecture under Salinity Stress

Root architecture and structure, and mineral homeostasis are closely linked with each other. The uptake and translocation of procured minerals toward other parts of trees is mainly based on root architecture [59]. Fruit size, i.e., weight, surface area, volume and length, is disturbed because of the adverse effects of saline conditions [60]. A significant decrease in biomass was also recorded in fruit crops growing under excessive salt levels. Roots have a basic contribution in water and mineral uptake. Minerals and water are necessary for sufficient growth and, subsequently, healthy trees [61]. An excess of Na^+^ and Cl^−^ accumulation in the root zone is toxic for fruit tree growth and yield. These can inhibit the uptake of K+ and numerous other essential minerals necessary for proper growth, yield and nutritional profiling [62]. Similarly, Walker et al. [63] also observed that the root system of trees acts as a reservoir for water, minerals and carbohydrates. 

Melatonin is found to be more efficient for mitigating the negative effects of abiotic stresses, especially salt stress. Improved root-related traits, i.e., biomass, length, surface area, volume and weight, were recorded even under salt stress environments in fruit trees [64]. An increase in macronutrient and micronutrient uptake was recorded following the exogenous application of melatonin because it was effective in improving the physiology of root traits [65]. The ionic balances were also recorded under exogenous spray of melatonin in those fruit trees growing under saline conditions [66]. The uptake of Na^+^ and Cl^−^ was found to be more balanced and uptake of K^+^ was improved due to the exogenous application of melatonin [67]. Melatonin had good capability to enhance the ion selectivity in the root zone. Root is considered the reservoir of mineral nutrients necessary for tree survival against harsh environmental stress [68]. Therefore, an improved root system is a basic need for increased tolerance in fruit trees. The root system also has the capability to inhibit the uptake and translocation of toxic ions to other tree parts [69]. Identification of tolerant/sensitive germplasm is clearly based on the root architecture of fruit trees and the ability of the root system to inhibit toxic ions from the soil [70].

Relative water potential, water-use competence and water transportation are enhanced by the exogenous use of melatonin [71]. Furthermore, water status within tree cell organelles and compartmentation can be enhanced through melatonin under salt stress [72].

### 2.4. Melatonin and Cuticle Formation in Leaves

Leaf cuticle is a multifunctional part of plants as it protects plants from different harsh climatic conditions such as temperature extremes, UV radiation, water deficits, mechanical deficits, insect–pest infestation and saline environments. The disturbances in leaf cuticle formation occur because of an excess of salts in the root zone of many fruit trees [73]. Exogenous application of melatonin improved formation of the leaf cuticle in fruit trees under salt stress conditions. It also reduced permeability and water loss, and delayed leaf wilting under saline and water-deficit conditions [73]. Permeability has a major contribution in the passage/blockage of solutes within plant cells and compartments. To overcome salinity stress, melatonin contributed a beneficial behavior to cope with adverse effects of salinity by improving formation of the leaf cuticle. 

### 2.5. Melatonin Enhances Shelf-Life of Fruits

The *Zizyphus* fruit is rich in vitamin C, with even higher levels than in oranges and kiwifruit. Its fruits contain 25–30% sugar, which is almost double the sugars found in common fruits and even greater than in sugarcane and sugar beet [74]. Compared with orange and kiwifruit, two well-known vitamin C-rich fruits, there was an expansion and high expression of genes involved in the biosynthesis and recycling of vitamin C, respectively [75]. There is a need for some management approaches to increase the shelf-life of fruits [76]. Melatonin is a pleiotropic molecule with multiple functions in fruit crops [77]. Therefore, it has been found to be more effective in increasing the shelf-life and decreasing postharvest decay in numerous fruit crops, i.e., peaches [78], strawberries [79], pears [80], cassava [81] and bananas [82]. Thus, its exogenous application is more effective for preservation of fruit crops. Melatonin at 100 μM L^−1^ enhanced the shelf-life of fruits with an improved plant defense system. The suppression of ethylene production was recorded in pears with exogenous application of melatonin. The beneficial impact of exogenous melatonin against abiotic stresses other than *Zizyphus* fruit crops is well described (Table 4). The impact of endogenous melatonin extracted from numerous fruit crops is also described (Table 5).

All plant species synthesize the indoleamine melatonin on their own. Many fruits and vegetables offer natural melatonin as a beneficial component of the diet. Melatonin functions not only as a signaling molecule but also as a potent free-radical scavenger and has a direct antioxidant effect. It is a safe and advantageous indoleamine [81]. Exogenous melatonin therapy has been proven to be a successful postharvest remedy for promoting ripening and improving tomato fruit quality, delaying postharvest senescence and increasing peach fruit-chilling resistance, attenuating postharvest decay and maintaining nutritive value of fruits under storage [83]. The impact of melatonin as a postharvest treatment on the postharvest quality of strawberry fruit has been shown; however, more work needs to be conducted on the postharvest physiology of minor fruit crops.

**Table 4:** Beneficial impact of exogenous melatonin against abiotic stresses in numerous fruit crops.

Fruit Crop Names	Cultivars	Stress Type	Concentrations	Functions	References
Banana	Williams	Drought	0, 40, 60 and 80 µM ppm	Melatonin administration found to be more efficient way for improved growth and yield under abiotic stresses. Melatonin treatments at 80 µM promoted growth and yield metrics.	[53]
Mango	Guifei	Salinity	0.5 mM	Melatonin interrupts the ripening and softening in mango fruit. Melatonin delays ethylene production and inhibits ethylene biosynthesis in mangoes.	[84]
Apple	*Malus hupehensis*	Salinity	0.1 µm	This is used as rootstock and this melatonin concentration is effective for improved growth and yield under saline conditions.	[85]
Grape	Muscat Hamburg	Control of postharvest losses	0.02, 0.2 and 2 mM L^−1^	It can activate defense responses to combat the infection of serious diseases such as *B. cinerea* in postharvest grapes.	[4]
Pistachio	Badami-Zarand	Salinity	0, 25, 50, 75, 100, 125 and 150 μM L^−1^	Exogenous application was also linked with higher increase in nutrient uptake. It can also attenuate the salinity damage via enhancing anti-oxidation ability, osmotic activity-adjustment and polyamine biosynthesis.	[86]

**Table 5:** Impact of endogenous melatonin extracted from numerous fruit crops.

Fruit Crop Names	Concentrations (ng/g)	Functions	References
Kiwifruit	0.02	Its concentration was measured in the seeds of some fruits and showed that melatonin concentration varied from leaves to seeds. However, improved endogenous melatonin increased the plant immunity to survive against adverse conditions.	[52]
Apple	0.05 and 0.16	Endogenous melatonin level was measured in the seeds of apples. The improved level of melatonin in seeds resulted in rapid germination of seeds with healthy growth.	[87]
Cherry	18.00	Bio-fortification of melatonin enhanced endogenous level of melatonin.	[88]
Banana	0.01, 0.47 and 0.67	Seed germination and seedling growth can be improved with application of melatonin because it is a multifunctional molecule discovered two decades ago.	[89]
Pomegranate	0.17	Higher endogenous melatonin concentration was recorded in medicinal crops compared with other crop.	[90]
Walnut	3.50	Phyto-melatonin is a multifunctional molecule found to be more effective for survival of plants against adverse climatic conditions	[91]

## 3. Melatonin Acts as a Defense against Salinity Stress Conditions

*Cand2* is an important binding protein present in plants endogenously. The biological functioning of *Cand2* is still unclear with regard to the proper functioning of these binding proteins for sustainable fruit production [92]. However, more investigation is required to study melatonin effects and its proteins in sustainable fruit production [8]. Melatonin has been recorded in numerous plant species. Different plant parts, i.e., seeds, roots, flowers and fruits, are rich sources of melatonin in different crops. The production level is found to be higher in the seed, while a lower concentration is estimated in fruits compared with other plant parts [93]. The Lamiaceae family is a rich source of this diverse multifunctional molecule, containing approximately 7110 ng g^−1^ [91]. Melatonin is recorded in the species Itaceae, Brassicaceae, Poaceae, Rosaceae and Rhamnaceae [94]. Many species also contain melatonin in higher amounts; however, there is possibility of hidden endogenous melatonin levels. Moreover, melatonin concentration chiefly depends on the type of species, environmental constraints, developmental stages and determination mechanisms, as studied by Byeon and Back [95] and Yi-feng et al. [96]. Huge variations are present in the endogenous concentration of melatonin within species [97]. Melatonin concentration was higher in the first two stages, then decreased at the third stage in two cultivars of cherry [98]. The role of melatonin in fruit developmental stages is still unclear. Melatonin can be effective at increasing the yield, quality and shelf-life of jujube fruits by improving the plant defense system. 

Melatonin is considered as a front-line soldier for those crops growing under salinity stress conditions [99]. Exploring the significance of melatonin is very imperative in minor fruit crops growing under harsh climatic conditions. Melatonin can be utilized in two ways: (a) agrochemical, and (b) by modified production of endogenous melatonin. Melatonin as an agrochemical can be applied to fruit crops in orchards exogenously. On the other hand, plants can be developed which produce modified concentrations of melatonin, improving the tolerance level against excessive salt levels [100]. The development of tolerance in fruit crops is imperative as it is a struggle to increase productivity to feed huge populations. However, it can be preferred only in controlled conditions. It is an effective approach, but traditional breeding requires much time and is laborious. The implications of different modern biotechnological tools can be explored for the development of resistance in the jujube germplasm against environmental stresses [91]. The exploration of melatonin will bring a revolution to the horticultural industry, especially for fruit crops. 

### 3.1. Crosstalk of Melatonin and Salinity Stress

**Figure 1:**
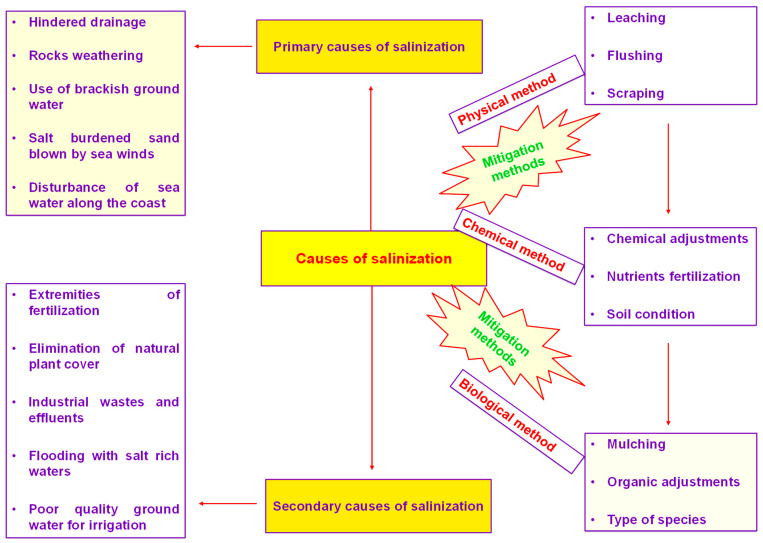
Causes of salt accumulation in soil and different mitigation methods to reduce salt concentrations.

**Figure 2:**
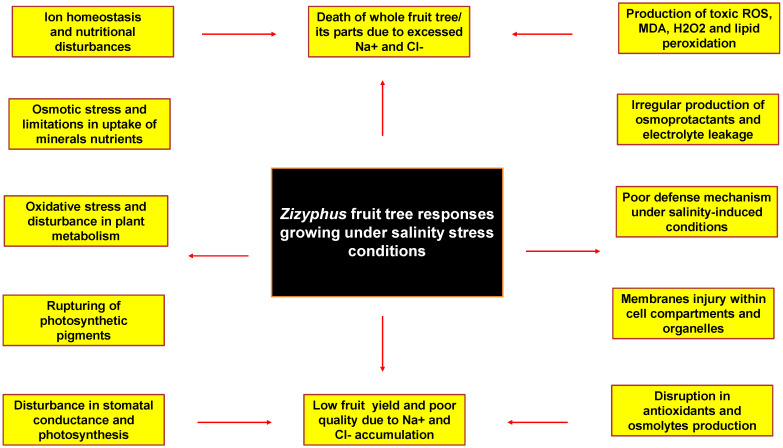
Exploration of biochemical and physiological responses occurring in Zizyphus fruit trees growing under saline conditions.

Salinity is drastically reducing fruit yield globally. Huge economic losses occur within the country, as reported by Suleyman et al. [101]. Salt stress conditions reduce the germination of seeds and emergence of seedlings. Excess of salt concentrations within the root zone is also a cause of osmotic stress conditions [102]. Different biochemical mechanisms, i.e., photosynthetic machinery, protein formation and lipid peroxidation, are disturbed under salinity-induced conditions, as studied by Li et al. [103]. Different management strategies, i.e., selectivity of ions and their exclusion, compartmentalization of ions, compatible solute synthesis, disturbance of photosynthesis pathways, alteration of membrane structures, antioxidant assays and osmolyte induction, gene expression and regulation, and phytohormones generation, can increase the fruit tree tolerance against adverse climatic conditions [104]. Causes of salt accumulation in soil and different mitigation methods to reduce salt concentrations are shown in Figure 1. Exploration of biochemical and physiological responses occurring in *Zizyphus* fruit tree growing under saline conditions are shown in Figure 2.

Exogenous application of melatonin is found be effective for reducing elevated growth inhibitors generated in fruits trees growing under elevated salinity conditions. The occurrence of oxidative injury is controlled by the application of melatonin [105]. Melatonin is one of the effective hormones for the elevation of salinity tolerance in fruit trees. The production of toxic ROS is regulated by melatonin application in fruit crops [106]. It is imperative for the scavenging of H_2_O_2_ and also decreases membrane injury by reducing lipid peroxidation [47].

Melatonin contributes to the catabolism of abscisic acid and biosynthesis of gibberellic acid. Moreover, upregulation of catabolism of ABA-related genes and down-regulation of the biosynthesis of ABA genes is required at the early germination stage, leading to good germination and excellent irreversible growth at initial stages [47]. Regulation of gene expression and upregulation of the tree defense system is maintained with exogenous application of melatonin [9]. The involvement of genes in nitrogen metabolism, carbohydrate metabolism, biosynthesis of hormones, overexpression of secondary metabolism, tricarboxylic acid transformation, metal handling and redox clearly showed the melatonin contribution in prompting metabolic activities [107].

### 3.2. Melatonin-Mediated Tolerance in Zizyphus

Fruit trees face multiple stress in a particular time duration. Underground and aerial tree parts are negatively affected because of the simultaneous incidence of single or more stresses. Grafting and non-grafting sense is also involved in salinity tolerance mechanisms because two diverse and compatible genotypes are united by graft union [108]. Rootstock is involved in the translocation of compatible solutes to other plant parts [109]. Higher accumulation of toxic ions was recorded in the root zone of fruit trees [110]. However, rootstock can restrict the transportation of toxic ions to other plant parts. Therefore, the plant signaling system is not solitary, but it links numerous transduction mechanisms in a complicated manner.

**Figure 3:**
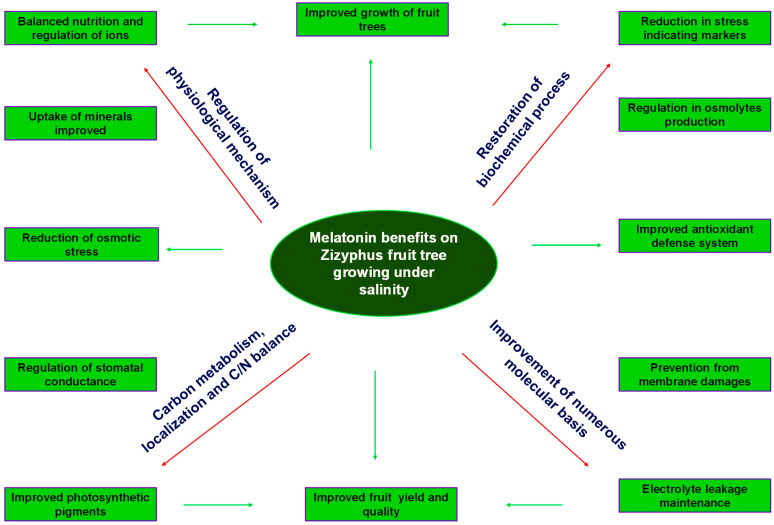
Melatonin is beneficial for restoration of biochemical and physiological responses occurring in Zizyphus fruit trees growing under saline conditions by improving the plant defense system.

Melatonin application is an essential approach to regulate different transduction methods against salinity conditions [111]. Melatonin primarily interferes with stress improvement and is a diverse tool containing different physiological and metabolic mechanisms. Deeper insights are required to unlock the molecular basis implemented by melatonin to elevate tolerance mechanisms against salt stress conditions [112]. Salinity stress decreases fruit tree growth, yield and quality of fruits. On the other hand, supplemental application of melatonin has the capability to cope with adverse effects on fruit tree growth, yield and quality by promoting excellent tolerance against salt stress [113]. There might be restrictions in the uptake of Na^+^/Cl^−^, and encouraging the uptake of mineral nutrients via roots is an effective way to cope with adverse effects of salinity in *Zizyphus* fruit crop by exogenous application of melatonin. Melatonin is considered a potential stress-releasing hormone that might be helpful for fruit trees to mitigate adverse effects of salinity [114]. Melatonin is beneficial for restoring biochemical and physiological responses occurring in *Zizyphus* fruit tree growing under saline conditions by improving the plant defense system (Figure 3).

Traditional breeding of fruit crops is very laborious and time consuming. Moreover, accuracy in traditional breeding is low cost but more inaccurate [1]. Application of modern biotechnological tools has had more efficacy than other traditional breeding. Higher heterozygosity and long juvenility are major problems in breeding *Zizyphus* germplasm [2]. Therefore, unlocking the potential of biochemical mechanisms, molecular approaches and proteomics are major factors that contribute to enhancing the salt tolerance mechanism of the *Zizyphus* germplasm. The identified salt-tolerant germplasm has the potential to mitigate adverse effects of salt stress [1], and production of modified melatonin within plant cells and compartments is effective for increased resistance in the *Zizyphus* germplasm. However, development of salt-tolerant genotypes using gene identification, genome mapping, genomic editing and genetic transformation is more imperative.

## 4. Melatonin and Mineral Uptake under Salinity Conditions

Proper growth, higher yields and superior fruit quality are mainly based on availability and utilization of mineral nutrients [115]. The disturbance in availability and translocation of mineral nutrients is dangerous for fruit tree health [8]. Salinity stress causes osmotic stress in fruit plants grown in salty soils. Higher salinity levels in the root zone of fruit trees induces osmotic stress conditions resulted in water deficit and nutritional imbalance situations [116]. Osmotic stress causes a reduction in the uptake of minerals, solutes and water content necessary for proper plant health. The reduction in translocation of macro- and micronutrients in fruit trees is disturbed due to osmotic stress, as reported by Ma et al. [117]. The uptake of minerals through roots is also disturbed due to the formation of bound nutrients because the unavailability of bound nutrients is also a major cause of nutrient deficiency in fruit trees [118]. Under salinity-induced conditions, it has been evaluated that uptake and translocation of minerals ratio root/shoot drastically decreases in the sour jujube [119]. 

Poor fruit yield and quality are due to mismanagement with regard to cultural practices, nutritional aspects, irrigation and climate change. Bombardment by chemicals is also toxic for soils and is a cause of compaction in soil. These poor soils are basic constraints which restrict the availability of mineral nutrients to fruit trees via roots [1]. For proper fruit size and its nutritional profiling, it is very important for exogenous application of melatonin to cope with the adverse effects of salinity [120]. The uptake, absorption and translocation of mineral nutrients from roots toward shoots and leaves were greatly improved with exogenous application of melatonin. Macro- and micronutrients were improved after exogenous application of melatonin [121].

The accumulation of Na^+^ and Cl^−^ in soil resulted in the translocation of these ions in higher concentrations and makes the genotypes more susceptible to stress conditions [122]. The tolerance mechanism was enhanced because of the inhibition of Na^+^ and Cl^−^ via roots. Sometimes, accumulated salts were absorbed in the rootstock and did not pass through to the scion/other tree parts [123]. Moreover, the identification and development of such tolerant rootstock had the potential to absorb toxic ions and restrict their translocation towards leaves, as reported by Juan et al. [124].

## 5. Melatonin Copes with Over-Generation of ROS, Lipid Peroxidation, H_2_O_2_ and MDA

Respiration (aerobic) is the cause of ROS production in all trees. ROS comprises different free radicals, e.g., hydroxyl radicals and anions of superoxide. On the other hand, non-radicals contain singlet oxygen and H_2_O_2_ [125]. A drastic reduction in O_2_ due to higher release of energy and electron transfer mechanisms indicates the over-generation of ROS within tree cells, organelles and compartments. Production sites of ROS are plasma membranes, mitochondria and chloroplasts [126]. These sites contribute in different cellular compartments of the respiration system [26]. Biotic and abiotic stresses are major causes of over-generation of ROS because of disturbances in cellular homeostasis within the fruit trees [127].

The toxic and beneficial level of ROS is mainly based on the type of species and its absorption rate. However, its optimum production is effective for the proper functioning of tree cells and organelles. Oxidative stress conditions occur because of over-generation of ROS. Higher concentrations of ROS are toxic for plant cells [26]. ROS generation is considered an oxidative stress marker because it is an important indicator of fruit trees growing under saline circumstances. ROS are famous as secondary messengers and act as intracellular signaling molecules as well as contribute in numerous reactions that occur within fruit tree cells [1].

Lipid peroxidation, MDA content and H_2_O_2_ are well-known stress-indicating markers in plants for detecting the stress conditions of trees. Their production is drastically enhanced when trees are subjected to stressful conditions, e.g., salinity, drought, heavy metals, mineral malnutrition, temperate extremes and pathogen attacks [128]. For the reduction of oxidative stress conditions, there is a need to balance the production and destruction conditions of ROS within tree cells. The increase of ROS from the optimum level resulted in increased membrane damage. The rupturing of membranes increases the harmful potential of oxidative stress conditions due increased concentration of lipid peroxidation within tree cells and their compartments [129].

Different major biomolecules, i.e., DNA, lipids and proteins, suffer production loss because of over production of ROS, MDA, H_2_O_2_ and lipid peroxidation under excessive saline conditions within the root zone of trees [130]. The rupturing of cells and poor functioning of biomolecules are because of higher concentration of these oxidative stress markers. Imbalanced ion and fluid transportation, hampered enzyme activation and disturbances in protein biosynthesis are due to oxidative stress conditions resulting in the loss of different plant cells/parts, or even death of the complete plant [130].

ROS generation is the first stress response when growing under saline conditions. Therefore, identification of salt-tolerant germplasm is more necessary for sustainable fruit production within the country. The development of tolerant germplasm is chiefly based on mitigation aspects of toxic ROS generation under salinity conditions [131]. 

Melatonin is an effective growth regulator involved in the mitigation of adverse effects of salinity in fruit trees for sustainable fruit production and superior quality with higher nutritional profiling [92]. It is effective in improving the ability to scavenge toxic ROS from tree cells, organelles and compartments. Overproduction of stress-indicating markers, i.e., ROS, lipid peroxidation, H_2_O_2_ and MDA, in fruit trees can be mitigated by exogenous application of melatonin [80]. Hence, *Zizyphus* fruit tree tolerance can be enhanced by exogenous application of melatonin.

## 6. Melatonin Activates the Fruit Tree Defense System

Oxidative stress conditions occur due to over-generation of ROS. The level of ROS overproduction is indicated by oxidative stress markers such as H_2_O_2_ and MDA activities. The production of enzymatic, non-enzymatic and different osmolytes is an effective strategy to cope with toxic ROS. These have excellent scavenging capability against toxic ROS by reducing membrane damage and protecting plants from oxidative stress injury. Adverse effects of salinity can include protein damage, cell membrane rupture, nucleotide disturbances, altered regulation of many enzymes and death of some cells, tissues and organs [132].

The plant defense system, which involves enzymatic, non-enzymatic and osmolyte production, regulates the overproduction of toxic ROS. These activities are important to balance ROS generation and their scavenging activities depend on a good immune system of the plant body. MDA, H_2_O_2_ and lipid peroxidation activities are reduced when there is balanced production of ROS because optimum production of ROS is effective for sustainable fruit production of horticultural crops, especially underutilized crops, as these are rich in essential nutrients and naturally hardy against harsh climatic conditions [133,134].

Some serious advancements are being used to improve oxidative stress resistance following the development of transgenic plants producing many antioxidants and osmolytes [135]. An antioxidant defense system is more powerful and supportive for the characterization of germplasm against salinity [136]. Furthermore, the plant defense process is also governed by the expression of different genes against salt stress conditions [137].

Fertilization is effective for the control of adverse effects that occurs from salinity in fruit trees. The production of different bioactive compounds is important for fruit tree survival against different environmental stresses [138]. Sufficient production of bioactive compounds results in an improved tolerance mechanism in fruit trees by improving the defense system, photosynthetic machinery, stomata conductance and respiration rate [139]. The maximum increase in antioxidant assays was recorded in tolerant germplasm, while the minimum was noted in sensitive germplasm of fruit crops under salinity stress conditions [98].

Melatonin is one of the emerging phytohormones necessary for sustainable fruit production in harsh climatic conditions [58]. Hence, it is considered very important to improve the defense system of fruit trees growing under excessive concentrations of salts, with the aim of better growth, higher yield and excellent fruit quality in numerous fruit crops [140], especially *Zizyphus* germplasm. The exposure of *Zizyphus* fruit trees to melatonin is very necessary because it is an underutilized fruit crop with excellent nutritional properties and hardy behavior against harsh climatic conditions. Therefore, supplemental application of melatonin can bring a greater revolution in yield and quality, not only in *Zizyphus* but also for numerous minor fruit crops. Melatonin increased the production of ROS scavengers by improving the plant defense system [141]. The tolerance in fruit trees against salt stress is based on activation of the defense system [91].

### Melatonin Regulates the Photosynthetic Mechanism

Numerous environmental stresses disturb the photosynthetic mechanism of plants. An excess of salts results in an imbalance in the photosynthetic process and carbon-reducing paths, and disturbances in the electron transport chain [142]. Chloroplast injury also occurs from salinity-induced conditions [143]. The disturbed metabolism is also due to rupturing of photosynthetic pigments. The rupturing of photosynthetic pigments is due to an excessive level of lipid peroxidation within plant cells and compartments [144]. The decreased water potential and low accumulation of necessary solutes results in osmotic stress. However, osmotic stress becomes more severe under water deficit conditions and interrupts the plant functioning in numerous ways, such as rupturing of the photosynthetic machinery [145]. Hence, fruit tree yield and quality are disturbed due to an excess of salts. The nutritional value of fruits is also reduced because of an excess of salts in the root zone.

Melatonin is found to be effective for fruit trees growing under saline conditions because of its higher antioxidant potential and stress-relieving property. Exogenous spray of melatonin revealed greater protection to fruit crops by restricting the damage that occurs in photosynthetic pigments (chlorophyll content, gaseous exchange and chlorophyll fluorescence), reducing oxidative stress conditions, activating the plant defense system and numerous other processes that occur within plant cells growing under saline conditions [80]. The content of *p*-coumaric acid was also reduced following foliar application of melatonin on fruit plants growing under saline environments [146]. Thus, it has been recorded that melatonin application is an important phytohormone for the regulation of photosynthesis, stomatal conductance and water potential [147,148].

## 7. Future Horizons

- Research on melatonin-mediated ripening of *Zizyphus* fruits under salinity-induced conditions is still elusive and much effort is required for further exploration. Crosstalk of melatonin with numerous other phytohormones can regulate different abiotic stresses, especially salt stress, in fruit crops such as the *Zizyphus* species.
- Alterations in the photo-pigment system and secondary metabolites as affected by exogenous melatonin levels against salt stress must be further explored. Accurate signaling, epigenetic paths and transcriptomic paths of melatonin still remain unknown and require more work on the *Zizyphus* fruit crop.
- It will be more mechanistic to explore the regulatory contribution of melatonin to alleviating stress tolerance and delivering distinctive immunity in *Zizyphus* fruit trees. This type of multifunctional phytohormone may possibly emerge as a sustainable alternative for regulating multiple stress responses in fruit trees.

## 8. Conclusions

The current study explored and provided detailed insights into the biochemical and physiological mechanism of *Zizyphus* to plant breeders for enhancing tolerance mechanisms against an excess of salts. *Zizyphus rotundifolia* cv. Gola was found to be more tolerant to growing under saline conditions due to an excellent regulating potential for photosynthesis processes and activation of plant defense systems. Osmolytes and secondary metabolic activities are the major factors for alleviation of tolerance against an excess of salts present in the root zone. Exploring the biochemical mechanism of *Zizyphus* will provide more support for the development of resistant germplasm to attain higher quality produce growing under saline environments.

## Figures and Tables

**Table 1:** Role of different antioxidant assays in salt-tolerance mechanism of *Zizyphus*.

Traits	Impacts	References
ROS	Its normal production is effective for normal functioning of plants. Its over-generation within cell compartments is indicative of stress conditions which are toxic for plants’ health.	[33]
MDA	Production of MDA content is indicative of stressed plants.	[34]
	Its reduction is effective for a decrease in lipid peroxidation of membranes that occurs due to an excess of salts.	[35]
	Higher salt-tolerance mechanism recorded through reduction in lipid peroxidation under salt stress.	[36]
H_2_O_2_	The production of H_2_O_2_ within plant cells and compartments is indicative of stress conditions faced by plants. Its scavenging is made possible by CAT activity naturally.	[37]
SOD	It is important to disturb the O_2_ to form H_2_O_2_ and remove the harmfulness of the superoxide anion.	[38]
POD	POD level is enhanced in *Z. spina-christi* under high salinity levels which contributes to scavenging of toxic ROS.	[38]
CAT	It mainly contributes to the reduction of H_2_O_2_ which is manufactured in light respiration in *Zizyphus* species.	[38]
APX	It also reduces the H_2_O_2_ generation in *Zizyphus* fruit species against osmotic stress conditions.	[39]
Glutathione	It is involved in maintaining normal cellular redox system of fruit plants either in normal or even in stressed conditions.	[40]
	H_2_O_2_ and its derivatives are quickly reduced through glutathione.	[41]
	It has excellent scavenging potential against salt stress.	[42]
Proline	Proline is considered an antioxidant that improves salt tolerance in *Zizyphus* plants.	[5]
	Proline may act as a signaling molecule in order to maintain osmotic regulation.	[43]
	Proline synthesis is largely increased in leaves and roots.	[43]
GB	It is very well known to regulate photosynthetic pigments and protein stability. Regulation of oxidative injury is necessary for higher yields. Gola cultivars of *Z. rotundifolia* are salt-tolerant and accumulate more glycine betaine than proline.	[44]
Photosynthetic pigments	Regulation of photosynthetic machinery is necessary for higher yields.	[45]
	Photosynthetic pigments rupture due to an excess of salts	[46]

ROS = reactive oxygen species; MDA = malondialdehyde; H_2_O_2_ = hydrogen peroxide; SOD = superoxidase dismutase; POD = peroxidase; CAT = catalases; APX = ascorbates and GB = glycine betaine.

**Table 2:** Fruit quality traits of *Zizyphus* as affected by salinity-induced conditions.

Traits	Functions	References
TSS	Decreased nutritional contents, such as TSS, are recorded in fruit trees under saline-induced environments.	[1]
AsA	It plays a significant role in reducing the hypoxia-induced oxidative injury in plants.	[47]
Phenolic content	Different phenolic compounds are present in fruit trees which mainly protect fruit trees from salinity stress by acting as a glucose-reservoir for osmoregulation and are essential constituents of the antioxidant defense mechanism.	[47]
Tocopherols	These have excellent potential to scavenge the excess toxic ROS and lipid radicals in plants. Lipid peroxidation is reduced due to production of tocopherols. These have greater potential to directly repair oxidizing radicals by inhibiting the chain transmission period during lipid auto-oxidation.	[48]
Flavonoids	Lipoxygenase production is restricted by generation of flavonoids; these also contribute well to improving plant defense system salinity stress conditions.	[49]
Different sugars	Reducing, non-reducing, and total sugars are drastically reduced due to the excess of salts within fruit tree cells and compartments.	[24]
TSP	Its concentration is decreased in leaves of *Zizyphus* fruit crop due to excessive salt concentrations within the plant cells.	[50]

TSS = Total soluble solids; AsA = ascorbic acid and TSP = total soluble protein.

**Table 3:** The critical concentration of salts that affect the fruit production in *Zizyphus* germplasm.

Species	Cultivars	Applied Concentrations	Duration	Threshold Concentrations	Effects of Salinity	References
*Z. jujuba* Mill.	Spinosa	50, 100 and 150 mM NaCl	0, 2, 6, 10, and 14 days	50 mM	Ploidy level can increase salt tolerance. Greater osmotic regulation was recorded in auto-tetraploidization than diploid germplasm of *Zizyphus*.	[11]
*Z. mauritiana* Lamk.	Gola and Umran	0, 4, 8, 12, and 16 dS m^−1^ of EC	After one day interval to avoid osmotic stress	4 dS m^−1^ of EC	Gola cultivar is more resistant against salt stress due to restoration of physiological and molecular basis.	[43]
*Z. mauritiana* and *Z. rotundifolia.*	Anonymous accessions at seedling stage	75 and 150 mM NaCl	4, 7 and 9 days	75 mM NaCl	Regulation of biochemical and physiological mechanisms due to strong defense system through 10 mg/L of uniconazole	[50]
*Z. jujuba* Mill.	Two years grafted seedlings of cultivar Jinsi-xiaozao	0, 3.0 and 5.0 g kg^−1^ applied in soil	Salt concentrations were applied on potted culture medium	3.0 kg^−1^ applied in soil	Photosynthetic pigments, i.e., chlorophyll a, b, total chlorophyll and Fv/Fo of PSII were reduced by 12.30% and 22.08%, respectively, compared with untreated plants	[51]
*Z. mauritiana* Lamk.	Dehli White, Suffon, Karella and Mehmood Wali	Brackish water 11 dsm^−1^ and 50% brackish water + 50% normal water	Every irrigation when required	50–60 mM	Dehli White cultivar of jujube is good for marginal lands in Pakistan because of good growth, yield and quality with improved defense system.	[6]
*Z. mauritiana* Lamk.	Banarsi Karaka, Narendra Ber Selection-1, Narendra Ber, Selection-2, Narendra Ber Selection-3, Pond and Gola	0, 4.0, 8.0, 12.0, 16.0 dSm^−1^ EC	Irrigation was applied alternately to ease the uniform dissemination of salts	0, 4.0 dSm^−1^ EC	The Na^+^ and Cl^-^ in leaves were enhanced due to increase in salinity. Jujube cvs. such as Banarsi Karaka, Narendra Ber, Selection-2 and Ponda can be placed in the tolerant group, and cvs. Narendra Ber Selection-1 and Gola as the semi-tolerant group. These findings may be supportive of commercial cultivation of jujube in salt-pretentious regions.	[52]
*Z. mauritiana* Lamk.	One year old budded plant of Umran cultivar	0, 50, 100, 150 and 200 meq 1^−1^ of NaCl, CaC1_2_, MgC1_2_ and MgS0_4_ were added in 1:1 (Na: Ca + Mg) and 3:7 (Cl:S04)	Regular irrigation was performed as per plant requirement	50 mM	Jujube, especially Umran, can be cultivated in salty lands with EC up to 11.30 dSm^−1^. This is the optimum level of EC at which jujube trees can be grown. The 50% yield reduction was recorded to be associated with a soil EC value of 11.30 dsm^−1^.	[47]
*Z. jujuba* Mill.	Dongzao	1 g L^−1^, 2 g L^−1^, 3 g L^−1^, 4 g L^−1^, and 5 g L^−1^	Regular irrigation was performed as per plant requirement	1 & 2 g L^−1^	Irrigation with low level of brackish water had little effect on the yield of winter jujube, but it reduced drastically after exceeding the threshold level of 3 g L^−1^.	[53]
*Z. Spaina-chrsity* (L.) and *Acacia tortillis* subsp. *tortillis*	*Zizyphus spina-christi* and *Acacia tortillis* subsp. *tortillis* seedlings	The mixed salts of Sodium and Calcium chloride (1:1 *v/v*) at concentrations of 1000–5000 ppm.	Regular irrigation was performed as per plant requirement	70 mM	*Acacia tortillis* subsp. *Tortillis* is found to be more tolerant compared with *Z. Spaina-chrsity* (L.)	[33]

## Data Availability

Not applicable.
